# Quantitative Evaluation of Collagen Crosslinks and Corresponding Tensile Mechanical Properties in Mouse Cervical Tissue during Normal Pregnancy

**DOI:** 10.1371/journal.pone.0112391

**Published:** 2014-11-14

**Authors:** Kyoko Yoshida, Hongfeng Jiang, MiJung Kim, Joy Vink, Serge Cremers, David Paik, Ronald Wapner, Mala Mahendroo, Kristin Myers

**Affiliations:** 1 Department of Mechanical Engineering, Columbia University, New York, New York, United States of America; 2 Department of Medicine, Columbia University Medical Center, New York, New York, United States of America; 3 Department of Opthalmology, Columbia University Medical Center, New York, New York, United States of America; 4 Department of Obstetrics and Gynecology, Columbia University Medical Center, New York, New York, United States of America; 5 Department of Obstetrics and Gynecology, UT Southwestern Medical Center, Dallas, Texas, United States of America; 6 Irving Institute for Clinical and Translational Research, Columbia University Medical Center, New York, New York, United States of America; Dalhousie University, Canada

## Abstract

The changes in the mechanical integrity of the cervix during pregnancy have implications for a successful delivery. Cervical collagens are known to remodel extensively in mice with progressing gestation leading to a soft cervix at term. During this process, mature crosslinked collagens are hypothesized to be replaced with immature less crosslinked collagens to facilitate cervical softening and ripening. To determine the mechanical role of collagen crosslinks during normal mouse cervical remodeling, tensile load-to-break tests were conducted for the following time points: nonpregnant (NP), gestation day (d) 6, 12, 15, 18 and 24 hr postpartum (PP) of the 19-day gestation period. Immature crosslinks (HLNL and DHLNL) and mature crosslinks (DPD and PYD) were measured using ultra performance liquid chromatography-electrospray ionization tandem mass spectrometry (UPLC-ESI-MS/MS). There were no significant changes in the total immature crosslink density (HLNL+DHLNL mol per collagen mol) throughout normal mouse gestation (range: 0.31–0.49). Total mature crosslink density (PYD+DPD mol per collagen mol) decreased significantly in early softening from d6 to d15 (d6: 0.17, d12: 0.097, d15: 0.026) and did not decrease with further gestation. The maturity ratio (total mature to total immature crosslinks) significantly decreased in early softening from d6 to d15 (d6: 0.2, d15: 0.074). All of the measured crosslinks correlated significantly with a measure of tissue stiffness and strength, with the exception of the immature crosslink HLNL. This data provides quantitative evidence to support the hypothesis that as mature crosslinked collagens decline, they are replaced by immature collagens to facilitate increased tissue compliance in the early softening period from d6 to d15.

## Introduction

During pregnancy, the cervix is the mechanical barrier that must remain closed until term, and must dramatically remodel into a compliant structure to allow for a successful term delivery. Abnormal cervical remodeling remains a significant clinical dilemma in obstetrics. Premature cervical remodeling is known to be a significant risk factor for preterm birth (PTB) [1]. Thus, in an effort to decrease the PTB rate attributable to premature cervical remodeling and to understand why some cervices fail to ripen at term, it is imperative that we first delineate the biochemical and mechanical properties involved in the spectrum of normal cervical remodeling.

In this study, we seek to understand the changes in collagen crosslinks with progressing gestation and how they relate to tensile mechanical properties of the cervix. The objectives of this study are: to identify and quantify the types of collagen crosslinks in the cervix and to determine the correlation between crosslinks and tensile mechanical properties of the tissue over the course of mouse pregnancy. We hypothesize that mature collagen crosslinks will decrease as mature collagen fibers are broken down in normal gestation to facilitate cervical remodeling. Additionally, we hypothesize that new collagens are synthesized during normal remodeling and thus the level of immature collagen crosslinks will increase in early pregnancy [2], [3]. We also hypothesize that samples with increased crosslink density, especially mature trivalent crosslinks, will have higher mechanical stiffness and strength [4], [5].

The cervix is known to be a highly collagenous structure. Fibrous collagens (types I and III) are the most abundant proteins in the extracellular matrix (ECM) and act as the structural support for the tissue. In a normal 19-day mouse gestation, the cervical ECM and tissue mechanical properties have been shown to change in four overlapping stages: softening, ripening, dilation, and repair [6], [7]. In early pregnancy (gestation day 1–12) the cervix progressively softens with measurable changes in tissue compliance by gestation day 12 (d12). During this softening phase, the cervical collagen solubility gradually increases [2] reaching maximum solubility around d12. From d12 to d18 the cervix continues to soften [8]. Before delivery on d19, the cervix dramatically softens, termed ripening, as the collagen fibers become highly dispersed as collagen fibril diameter and inter-fibrillar spacing increases [3]. At d19, the cervix reaches its maximum tissue distensibility and dilates concurrently with uterine contractions for delivery. In postpartum, the cervix undergoes a repair process in which the cervical mechanical properties and collagen fibers eventually return to its nonpregnant state.

Enzymatic intermolecular crosslinks are known to stabilize collagen molecules and help organize them into hierarchical structures. In connective tissues, there are two pathways for enzymatic collagen crosslinks: allysine and hydroxyallysine pathways which generally occur in loose and stiff connective tissues respectively. Pyridionline crosslinks are derived from the hydroxyallysine crosslink pathway and have been shown to occur in other connective tissues [9]. In this crosslink pathway, crosslinks are initially formed between a telopeptide residue and a helical residue to produce immature (divalent) crosslinks including dehydro-dihydroxylysinonorleucine (deH-DHLNL) and dehydro-hydroxylysinonorleucine (deH-HLNL) [10]. These immature crosslinks react with another telopeptide residue to form mature (trivalent) crosslinks between three collagen molecules to form deoxypyridinoline (DPD) and pyridinoline (PYD). In this study, we use ultra performance liquid chromatography-electrospray ionization tandem mass spectrometry (UPLC-ESI-MS/MS) to measure DHLNL (reduced form of deH-DHLNL), HLNL (reduced form of deH-HLNL), pyridinoline (PYD, also known as hydroxylysyl-pyridinoline) and deoxy-pyridinoline (DPD, also known as lysyl-pyridinoline), hydroxyproline (OHP) for total collagen content at different gestation time points in a normal mouse pregnancy. We have previously shown that PYD values remain elevated in a mouse model with a parturition defect due to disrupted cervical remodeling [11], and we have shown that DHLNL, PYD, and DPD crosslinks exist in nonpregnant human cervical tissue [12].

Biochemical studies show that the extractability of collagen in weak acids increase significantly with progression of pregnancy in the mouse without significant changes in the total collagen content per dry weight [2]. We have demonstrated similar results on human nonpregnant and term pregnant tissue samples [13]–[15]. Since weak acids are able to solubilize collagens with reduced crosslinks [16], tissues with high extractability indicate less crosslinked collagens. Histological studies on cervical tissue show changes in collagen ultrastructure from aligned fiber arrangements with defined directionality in nonpregnant tissue to dispersed fibers with less defined orientations in pregnant tissue in both humans [13], [17] and mice [18]. These results together suggest that in normal cervical remodeling, older more crosslinked collagens are simultaneously broken down and replaced by new less crosslinked collagen, leading to an overall softer tissue without changes in the overall collagen content.

The breakdown of mature crosslinked collagens in the mouse cervix have been evaluated quantitatively in a study by Akins et al. [3], where they showed that the mature crosslinks PYD and DPD decreased significantly with pregnancy. These results directly indicate the breakdown of older collagens during cervical remodeling. Immature crosslinks and the ratio between mature to immature crosslinks give a measure of collagen production and collagen maturity [10]. Immature crosslinks have also been tied to physiological function in other tissues such as bone [19], and have not been previously quantified for in the mouse cervix. A study in developing embryonic tendon demonstrated that inhibiting the formation of crosslinks through a disruption of the enzyme lysyl oxidase (LOX) led to a reduced elastic modulus in the tissue [4]. In the cervix, LOX activity has been shown to decrease in early pregnancy [20]. Taken together, these evidence demonstrate the importance of measuring both immature and mature collagen crosslinks as well as corresponding cervical mechanical properties. Therefore, in this paper we report the amount and types of collagen crosslink in mouse cervical tissue, the maturity of collagen in the tissue at a given gestational age, and the role of crosslinks with mechanical properties.

## Materials and Methods

### Mouse cervical samples

Animals were housed under a 12L:12D photoperiod (lights on 0600-1800) at 22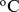. Mice used in the present studies were from Black 6/129 SvEv strain. These mice were generated and maintained as a breeder colony at the University of Texas Southwestern Medical Center (Dallas, TX). Female mice were housed overnight with males and checked in the morning for vaginal plugs to obtain accurately timed pregnant mice. The day of plug formation was counted as day 0, and birth occurred in the early morning hours of d19. Samples were collected at midday except for gestation day 18, which were collected in the evening of d18, between 1800 and 2000. All mice in these studies were 3–6 months old and nulliparous. All studies were conducted on approval by the University of Texas Southwestern Medical Center Institutional Animal Care and Research Advisory Committee. Animals were anesthetized with Avertin and then sacrificed via cervical dislocation. Reproductive tracts were collected from nonpregnant, gestation day 6, 12, 15, 18, and 24 hr postpartum mice and immediately frozen (n = 3 to 8 per gestation group). Samples were shipped to Columbia University on dry ice and stored at 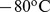 until testing.

### Mechanical Testing

**Figure 1:**
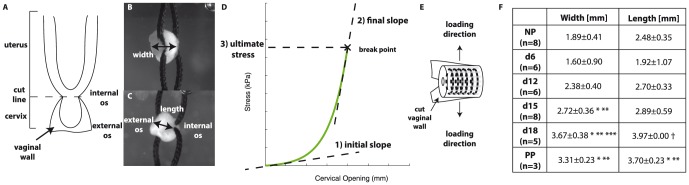
Mechanical testing of cervical samples. A) Diagram of a mouse reproductive tract. Mechanically tested samples were cut at the cutline indicated with the vaginal wall attached. Camera images of sutured cervical samples for tensile testing demonstrating measured geometry B) looking at external os of cervix with width measurement and C) looking along the length of the cervix and length measurement. D) Diagram demonstrating mechanical property parameters calculated for each sample. x indicates the breaking point where the sudden drop in load indicated failed sample. E) Diagram demonstrating circumferentially oriented fibers and loading direction parallel to fibers. F) Table of sample dimensions for each gestation group. *, **, *** represents statically significant difference compared to NP, d6, and d12 respectively. †represents statically significant difference compared to all other groups.

To investigate the correlation between intermolecular collagen crosslinks and cervical mechanical properties, tensile tests were conducted on samples from each of the designated gestational age groups. For mechanical testing, the vaginal tissue was left attached to the cervix for consistency purposes. Here, the load is carried by the cervix because the vaginal wall was split perpendicular to the loading axis (Fig. 1E). Frozen samples were thawed in phosphate buffered saline (PBS) and cervical tissue was detached from the uterus at the junction of the uterine horns (Fig. 1A). Two surgical sutures (Perma-Hand Silk 2-0, Ethicon) presoaked in PBS were passed through the cervical canal and the undeformed width and length were measured (Fig. 1B,C). Sutures were attached to custom tensile grips on a universal testing machine (Model 5948 MicroTester, Instron, 50N load cell) and immersed in PBS (Fig. 1B,C) throughout testing. After adding a small preload (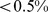 of maximum load) samples were pulled in tension at 0.1 mm/s until break, while recording force [N] and displacement [mm]. Stress was calculated using the dimensions obtained before testing and loading curves (cervical opening [mm] vs stress [kPa]) were generated. These loading curves were assessed to determine the initial slope [kPa/mm], final slope [kPa/mm] and breaking strength [kPa] (Fig. 1D). After mechanical testing, samples were stored at 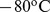 until processing for crosslink analysis.

### Sample Preparation for Collagen Crosslink Analysis

Vaginal tissues surrounding the cervix were carefully removed from mechanically tested samples (n = 3 to 8 per gestational group). All samples were flash frozen in liquid nitrogen, lyophilized and weighed to determine its dry weight. Sodium borohydride (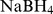) was added to each sample as a reduction step for the divalent crosslinks (HLNL and DHLNL). Samples were treated with acetic acid and brought to pH 4 to stop the reduction reaction, washed with distilled water, and hydrolyzed under vacuum at 6N hydrochloric acid (HCl) for 18–24 hours at 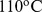. The hydrolysate from each sample was resuspended in heptafluorobutyric acid (HFBA) and lyophilized overnight. The reduced, hydrolyzed, lyophilized samples were resuspended in 20
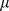
l of distilled water and transferred to the Biomarkers Core Lab of the Irving Institute for Clinical and Translational Research for ultra performance liquid chromatography-electrospray ionization tandem mass spectrometry (UPLC-ESI-MS/MS) analysis [21].

### Ultra Performance Liquid Chromatogrphy-Electrospray Ionization Tandem Mass Spectrometry (UPLC-ESI-MS/MS)

The immature crosslinks dihydroxylysionorleucine (DHLNL) and hydroxylysinonorleucine (HLNL), the trivalent mature crosslinks pyridinoline (PYD) and deoxypyridinoline (DPD), as well as hydroxyproline (OHP) to determine total collagen were quantified in the prepared samples by ultra performance liquid chromatography-electrospray ionization tandem mass spectrometry (UPLC-ESI-MS/MS) using a method adapted from [21]–[24] and as previously described [11]. The method, which consists of two sequential UPLC-ESI-MS/MS assays, is able measure PYD, DPD, DHLNL, HLNL, and OHP in a single sample. The sample pretreatment without a reduction step precluded the measurement of the divalent crosslink DHLNL and HLNL.

In short, calibration standards pyridinoline (PYD), deoxypyridinoline (DPD) and internal standard actetylated pyridinoline (AcPYD) were purchased from Quidel Corp. (San Diego, CA, USA). Dihydroxylysinonorleucine (DHLNL) was purchased from Santa Cruz Biotechnology (Santa Cruz, CA, USA). Hydroxylysinonorleucine (HLNL) was obtained as a kind gift from Professor Simon P. Robins from the Rowett Institute of Nutrition and Health, University of Aberdeen, Scotland, United Kingdom. Hydroxyproline (OHP) was purchased from Sigma-Aldrich. Deuterated hydroxyproline (hydroxyproline-D3) was purchased from C/D/N Isotopes Inc (Pointe-Claire, Quebec, Canada). Heptafluorobutyric acid (HFBA) grade water and acetonitrile and other common chemicals were purchased from Fisher Scientific (Pittsburgh, PA, USA) or Sigma-Aldrich (St. Louis, MO, USA). All assays were carried out on a Waters Xevo TQ MS ACQUITY UPLC system (Waters, Milford, MA, USA). The system was controlled by MassLynx Software 4.1. The sample hydrolysate was reconstituted in 20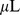 of 2% HFBA solution containing 2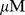 AcPYD as internal standard and throughly vortexed. The sample was centrifuged at 12,000 g for 15 min at 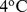 and clear aqueous phase was transferred to an Agilent clear screw top micro sampling LC/MS vial (P/N 5184-3550. Agilent Tech, Santa Clara, CA, USA) for UPLC/MS/MS assay of collagen crosslinks [22], [23]. The sample was maintained at 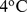 in the autosampler and a volume of 5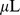 was loaded onto a ACQUITY UPLC HHS C18 column (2.1 mm inner diameter ×100 mm with 1.8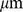 particles, Waters, P/N 186003533), and a 2.1×5 mm guard column with the same packing material (Waters, P/N 186003981). The column was maintained at 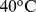. The flow rate was 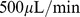 in a binary gradient mode with the following mobile phase gradient: initiated with 90% phase A (water containing 0.12% HFBA) and 10% mobile phase B (acetonitrile containing 0.06% HFBA). The gradient of acetonitrile was increased linearly to 35% over 4 min, then to 95% in 0.2 minute and maintained for 1 more minute. The column was subsequently conditioned by using the initial gradient for 1 minute after which the next sample was injected. After injection, 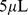 from the remaining sample was transferred to another LC/MS vial and diluted with 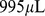 of water containing 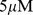 of hydroxyproline-D3 as internal standand for hydroxyproline assay [24]. The sample was vortexed well and 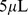 was injected to a Waters ACQUITY UPLC BEH Phenyl column (3 mm inner diameter ×100 mm with 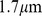 particles, Waters, P/N 186004673), preceded by a 2.1×5 mm guard column containing the same packing (Waters, P/N 186003979). The column was maintained at 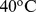. The flow rate was 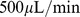 in a binary gradient mode with the following mobile phase gradient: initiated with 99% phase A (water containing 0.1% formic acid) and 1% mobile phase B (acetonitrile containing 0.1% formic acid). The gradient of acetonitrile was increased linearly to 50% over 5 min, then to 95% in 0.2 minute and maintained for 1 more minute. The column was then conditioned by using the initial gradient for 1 minute and the next sample was injected. Positive ESI-MS/MS with multiple reaction monitoring (MRM) mode was performed in all the assays using the following parameters: capillary voltage 4 kV, source temperature 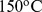, desolvation temperature 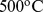, desolvation gas flow 1000 L/hr. Optimized MRM parameters are listed in Table 1.

**Table 1:** Optimized MRM conditions.

Compound	MRM transition (m/z)	Cone voltage (V)	Collision Energy (eV)
Hydroxyproline	132.09 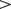 86.07	20	14
Hydroxyproline-D3	135.11 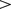 71.04	20	28
HLNL	292.2 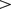 84.0	26	28
DHLNL	308.2 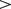 128.1	31	22
PEN	379.2 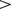 135.0	40	40
DPD	413.2 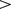 267.1	44	28
PYD	429.2 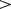 267.1	44	28
AcPYD (IS)	471.2 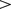 267.1	44	28

### Calculation of Collagen Crosslink Densities and Collagen Content

Total collagen content for each sample was determined by assuming 14% OHP content per collagen by weight [21] then normalized by tissue dry weight [mg/mg]. Collagen crosslink densities of the tissue were calculated by dividing the concentration of HLNL, DHLNL, DPD, and PYD by the concentration of collagen on a mole to mole basis [mol/mol]. The density of total immature crosslinks were determined by adding HLNL and DHLNL densities. The density of total mature crosslinks were determined by adding DPD and PYD densities. The maturity ratio was determined by calculating the ratio between total mature (DPD+PYD) to total immature (HLNL+DHLNL) crosslinks.

### Statistics

Differences in the collagen crosslink densities and mechanical properties between gestation groups were analyzed using one way analysis of variance (ANOVA). In order to determine differences between any two groups, Tukey's honestly significant difference criterion was used with MATLAB's Statistics Toolbox (v8.1.0.604, Natick, MA). To determine the significance of the correlation between crosslink densities and mechanical properties, a simple linear regression was fit to the data with crosslink density as the explanatory variable. Significant differences and correlations were determined for 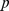-values less than 0.05 for all statistical analysis.

## Results

### Tensile mechanical properties

**Figure 2:**
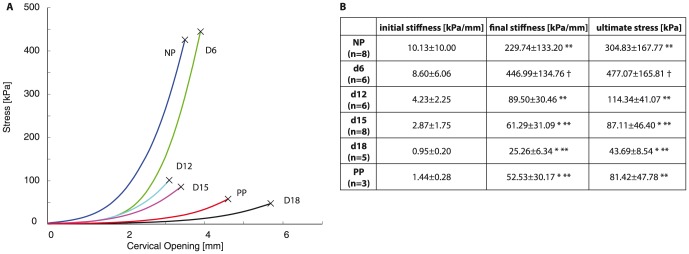
Cervix mechanical stiffness and strength decreases with advancing gestation. A) Representative cervical opening vs stress curves for normal cervix with progressing gestation and postpartum with each color representing each gestation day group. Legend: NP - blue, d6 - green, d12 - cyan, d15- magenta, d18 - black, PP - red; n = 3 for each group. B) Table of mechanical properties calculated from mechanical tests with average values 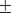 standard deviation. * and ** represents statically significant difference compared to NP and d6 respectively. †represents statically significant difference compared to all other groups.

The tensile mechanical response of the cervix changed drastically from a stiff structure into a soft structure as pregnancy progressed (Fig. 2A), which is consistent with previous studies [8]. On average, initial stiffness (slope of the line fit to the initial linear region of loading curve) decreased gradually without significant difference between groups. The final stiffness (slope of the line fit to the linear region of the loading curve before breaking) and the ultimate stress (maximum stress sustained by the tissue before breaking) increased significantly from NP to d6, decreased significantly from d6 to d12 and continued to decrease until d18 (Fig. 2).

### Collagen crosslink densities

**Figure 3:**
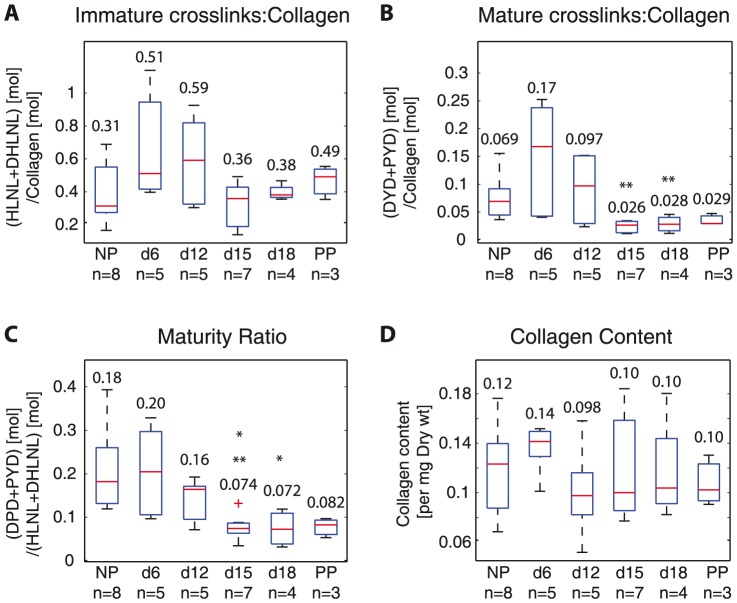
Collagen crosslinks increase in early pregnancy and decrease by late gestation, leading to decreased maturity ratios in late pregnancy. Averaged crosslink density (normalized by collagen) measured with UPLC-ESI-MS/MS for all samples for A) total immature crosslink density (HLNL+DHLNL) B) total mature crosslink density (DPD+PYD), C) maturity ratio (total immature crosslink density: total mature crosslink density), and D) collagen content (per dry weight). * and ** represents statically significant difference compared to NP and d6 respectively. (One-way ANOVA 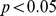). NP  =  nonpregnant, d6, d12, d15, d18  =  gestation day 6, 12, 15, 18, PP  =  postpartum. Numbers on top or below boxes are median values for each group.

Immature crosslinks remained unchanged while mature crosslinks decreased during the early softening stage (d6 to d12 to d15). Total immature crosslink density (HLNL+DHLNL) increased from NP to d6, but not significantly (NP: 0.31 to d6: 0.51) and they decreased between d12 and d15, but not significantly (d12: 0.59 vs d15: 0.36). At d15, d18, and PP the density values (range: 0.36–0.49) were not significantly different from NP levels (0.31) (Fig. 3A). Total mature crosslink density (DPD+PYD) increased from NP to d6, but not significantly (NP: 0.069, d6: 0.17) and decreased significantly from d6 (0.17) to d12 (0.097) to d15 (0.026). Total mature crosslink density levels at d15, and d18 (range: 0.026–0.028) were significantly lower than NP levels (0.069) (Fig. 3B).

There were no significant differences in the maturity ratio between NP (0.18) and d6 (0.20) (Fig. 3C). There was a significant drop in maturity ratio from d12 (0.16) to d15 (0.074), indicating a shift to less crosslinked, immature collagens by d15 (Fig. 3C). The maturity ratios from d15 and d18 were significantly lower compared to NP levels. There were no significant differences in the collagen content between all gestation groups (Fig. 3D).

**Figure 4:**
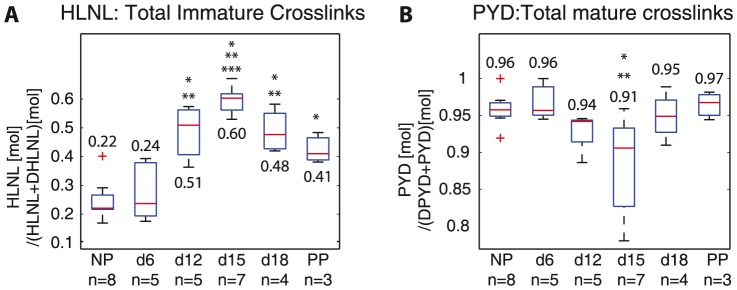
Dominance of HLNL and DHLNL on total immature crosslink density reverse between d6 and d15. PYD remains dominant over DPD on total mature crosslink density throughout gestation Ratios of individual crosslinks on total immature and mature crosslinks: A) HLNL:(HLNL+DHLNL) and B) PYD:(DPD+PYD). *, **, and ** represents statically significant difference compared to NP, d6, and d12 respectively. (One-way ANOVA 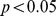); n = 6 (mechanically tested (n = 3) and non-mechanically tested (n = 3)) are combined and averaged within each gestation day group. NP  =  nonpregnant, d6, d12, d15, d18  =  gestation day 6, 12, 15, 18, PP  =  postpartum. Numbers on top or below boxes are median values for each group.

To determine the contribution of HLNL, DHLNL, DPD, and PYD to the total immature and total mature crosslinks in pregnancy, respectively, we calculated the ratio of HLNL and DHLNL to total immature crosslink density and ratio of DPD and PYD to total mature crosslink density (Fig. 4A,B). The contribution of HLNL and DHLNL on the total immature crosslink density reversed in pregnancy, while PYD and DPD ratios remained steady throughout gestation. There was a shift from DHLNL dominance in NP and d6 samples of total immature crosslink density (HLNL: 22–24% vs DHLNL: 76–78%) to even ratios of HLNL and DHLNL at d12, HLNL dominance in d15 (HLNL: 60% vs DHLNL: 40%), back to even ratios in d18 and PP. For the mature crosslinks, PYD remained dominant over DPD throughout pregnancy (DPD: 4–9% vs PYD: 91%–96%).

### Relationships between collagen crosslink densities and mechanical properties

**Figure 5:**
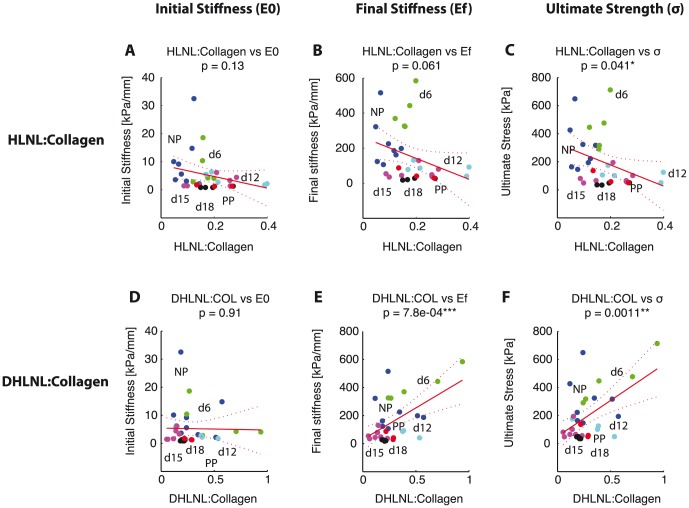
DHLNL immature crosslink density correlates with mechanical properties, but not HLNL. Scatter plots showing correlation between immature crosslink densities and mechanical properties. HLNL density on the horizontal axis versus: A) initial stiffness, B) final stiffness, and C) ultimate stress. DHLNL density on the horizontal axis versus: D) initial stiffness, E) final stiffness, and F) ultimate stress. Colors represent gestation groups: NP - blue, d6 - green, d12 - cyan, d15 - magenta, d18 - black, and PP - red. Red line represents simple linear regression line between crosslink density and each mechanical property. Dotted lines represent 95% confidence interval. The 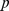-values for regression listed for each correlation with number of *'s representing significance.

**Figure 6:**
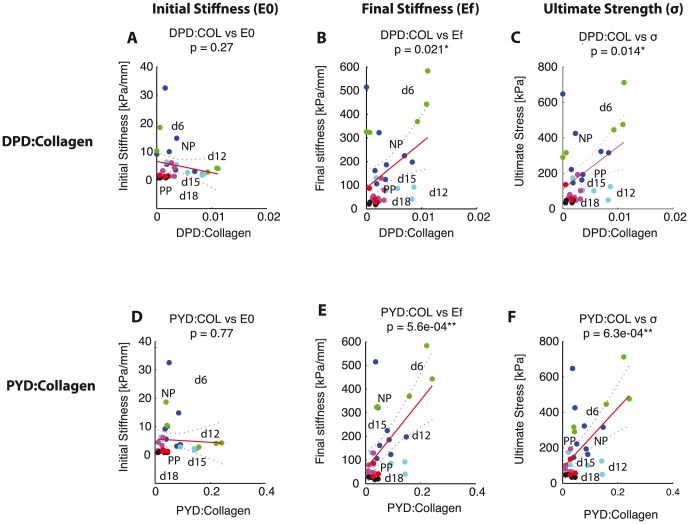
Both mature crosslink density correlate with mechanical properties. Scatter plots showing correlation between mature crosslink densities and mechanical properties. DPD density on the horizontal axis versus: A) initial stiffness, B) final stiffness, and C) ultimate stress. PYD density on the horizontal axis versus: D) initial stiffness, E) final stiffness, and F) ultimate stress. There was a stronger correlation for PYD crosslink density with mechanical properties. Colors represent gestation groups: NP - blue, d6 - green, d12 - cyan, d15 - magenta, d18 - black, and PP - red. Red line represents simple linear regression line between crosslink density and each mechanical property. Dotted lines represent 95% confidence interval. The 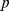-values for regression listed for each correlation with number of *'s representing significance.

**Figure 7:**
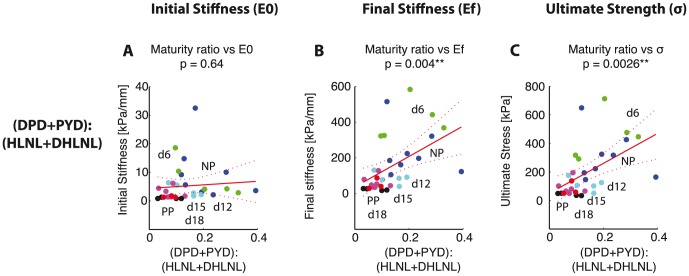
Maturity ratio correlates with mechanical properties. Scatter plots showing correlation between maturity ratio (DHLNL+HLNL):(DPD+PYD) and mechanical properties. Maturity ratio on the horizontal axis versus: A) initial stiffness, B) final stiffness, and C) ultimate stress. Colors represent gestation groups: NP - blue, d6 - green, d12 - cyan, d15 - magenta, d18 - black, and PP - red. Red line represents simple linear regression line between crosslink density and each mechanical property. Dotted lines represent 95% confidence interval. The 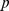-values for regression listed for each correlation with number of *'s representing significance.

**Figure 8:**
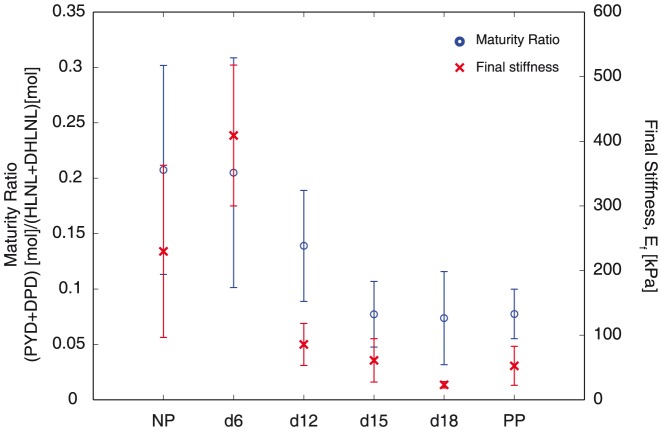
Evolution of collagen crosslinks correlate to mechanical properties in normal mouse gestation. Plot showing the changes in both mature collagen crosslinks and mechanical properties (final stiffness) with progressing gestation. Blue circles represent total mature crosslinks (on the left y-axis) and red x's represent final stiffness (on the right y-axis) as a function of gestation on the x-axis.

Collagen crosslink densities (DHLNL, DPD, PYD) and maturity ratio correlated significantly with tissue tensile mechanical properties. We fit a simple linear regression model to the data with the crosslink density as the explanatory variable. For immature crosslinks, there was no significant correlation between HLNL crosslink density with initial and final stiffness (Fig. 5A,B 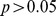). HLNL crosslink density was negatively correlated to ultimate strength (Fig. 5C 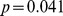). DHLNL crosslink density was significantly and positively correlated to final stiffness and ultimate strength (Fig. 5E,F, 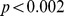). For mature crosslinks, both DPD and PYD density showed significant positive correlations with final stiffness and ultimate strength with 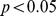. PYD showed more significant correlations to both mechanical properties in comparison with DPD (Fig. 6B,C,E,F). Despite the lack of correlation between HLNL and mechanical properties, the maturity ratio showed significant correlation with mechanical properties (Fig. 7). The correlation between maturity ratio and final stiffness is summarized and presented a function of progressing gestation in Fig. 8.

**Figure 9:**
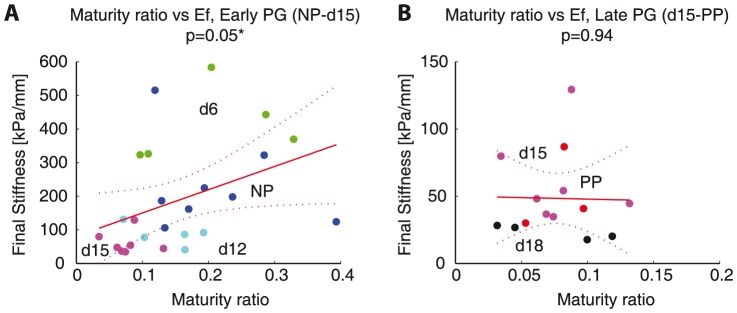
Significant correlations between maturity ratio and mechanical properties in early pregnancy (NP-d15) but not in late pregnancy (d15-PP). Adaptation of Figure 7B. A) Maturity ratio vs final stiffness for NP-d15, B) maturity ratio vs final stiffness for d15-PP. Colors represent gestation groups: NP - blue, d6 - green, d12 - cyan, d15 - magenta, d18 - black, and PP - red. Red line represents simple linear regression line between crosslink density and final stiffness. Dotted lines represent 95% confidence interval. The 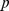-values for regression listed for each correlation with number of * representing significance (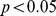).

The significant correlations between crosslinks and mechanical properties presented are in part due to the parallel occurrence of decreasing collagen crosslinks and increasing tissue compliance across the gestation timeline. In Fig. 9, we separated the scatter plot shown in Fig. 7B of maturity ratio vs final stiffness between NP, d6, d12, d15 (Fig. 9A) and d15, d18, and PP (Fig. 9B) and fit separate linear regression models to each plot. Here we see that maturity ratio is significantly and positively correlated to final stiffness in early softening (NP, d6, d12, d15) but not in late softening (d15, d18, PP) when separated into these two groups. This stronger correlation in the early softening group suggests that collagen crosslinks have a mechanical role in early softening, while their role and interactions with other ECM components in late softening still remain to be determined.

## Discussion

The objectives of this study are to determine the changes in immature and mature collagen crosslinks in a normal mouse pregnancy and to determine its correlations to tissue mechanical properties. Both total immature and total mature collagen crosslink densities increase in early pregnancy (NP to d6) and decrease significantly between d12 and d15 (Fig. 3,A-C). The contributions of DHLNL and HLNL to the total immature crosslink densities shift during pregnancy, with more DHLNL in NP and d6 tissue, even levels in d12, more HLNL in d15 tissue, and even levels in d18 and PP. PYD is dominant over DPD in contributions to total mature crosslink density throughout gestation (Fig. 4). An increase in all crosslinks (except for HLNL) results in an increase in final stiffness and ultimate strength (Fig. 5,6). Maturity ratio (DPD+PYD):(DHLNL+HLNL) correlates significantly with mechanical properties (Fig. 7).

The crosslink density and collagen content results were in agreement with data presented in [3], measured through high performance liquid chromatography (HPLC) methods and colorimetric assay respectively. The changes in the immature and mature crosslinks during cervical remodeling lead to overall decreases in both crosslink maturity ratios during pregnancy. This shift in the maturity ratios is quantitative evidence to support our hypothesis of collagen turnover during pregnancy from mature highly crosslinked collagens to immature less crosslinked collagens (Fig. 3E,F). The correlations between crosslink densities and mechanical properties support our hypothesis that more crosslinked collagen, especially mature crosslinks correlate with stiffer, stronger tissue properties (Fig. 5,6, 9). Both the immature DHLNL crosslink and mature PYD crosslink exhibit stronger correlations with mechanical properties as evident by the smaller 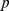-values compared to the mature DPD crosslink. These results suggest that DHLNL and PYD crosslinks play an important role in tissue mechanical properties. Additionally, we found that DHLNL and HLNL dominance on the total immature crosslinks changes throughout gestation. Decreasing dominance of DHLNL density levels correlated to increasing tissue compliance.

In the results presented here, we see stronger correlations between crosslinks versus the final stiffness and ultimate strength compared to the initial stiffness of the tissue. These correlations suggest that crosslinks have a role in determining the stiffness and strength of fully straightened collagen fibers in the cervix. These results are consistent with findings from studies on other collagenous tissues. One study on connective tissues in immature bovine knee joint showed that PYD crosslink density played a preferential role over collagen content in determining tensile stiffness for certain tissues [5]. Another study on developing embryonic tendon showed that inhibiting LOX mediated crosslinking, which includes the crosslinks investigated in this study, led to reduced tissue elastic modulus without affecting collagen morphology or content [4]. Additionally, our results are in agreement with molecular multi-scale models of crosslinked collagen fibrils, which show that increasing collagen crosslink densities lead to fibrils with greater mechanical strength and stiffness in large deformation [25]. The correlations between crosslinks and mechanical properties presented here along with these studies suggest that intermolecular crosslinks are important in determining tissue mechanical properties. However, the results here suggest that the breakdown of crosslinks facilitate cervical softening in the early softening stage (NP to d15), but not in the late softening stage (d15 to d18) nor ripening (Fig. 8). There are no clear differences in any of the crosslinks or maturity ratios in late softening between d15 and d18. Although the differences in the mechanical parameters measured here are not significant between d15 and d18, the loading curves (Fig. 2) and previous studies [8] demonstrate that the tissue continues to increase in compliance in this late softening period. These results give motivation to study other matricellular proteins and ECM components that contribute to the mechanical changes that occur during late softening and ripening.

### Clinical Implications and Future Studies

In this study, we established the evolution of immature and mature crosslinks during normal cervical remodeling in mice. One of the challenges in treating premature cervical remodeling in human pregnancies is the fact that we still do not understand the pathophysiology behind the condition. The data presented here suggest that during the cervical softening phase in mice, shifts in the collagen crosslinks occur before d15. As such, we are currently evaluating if there are differences in the collagen crosslink profiles between cervical tissue from women with a history of premature cervical remodeling compared to women with normal cervical remodeling. This study will help elucidate if collagen crosslinks have a role in explaining the cervical changes that occur with this condition. In addition, there are *in-vivo* tools currently in development to diagnose premature cervical remodeling including the collascope, which uses the fluorescence of PYD to measure its concentration [26], [27], second harmonic endoscope, which can image the ultrastructure of collagen [17], and Raman spectroscopy, which has the potential to detect changes in different ECM components including collagen crosslinks [28]. The data presented here, together with future developments in these tools have the potential to detect premature cervical remodeling earlier in pregnancy so interventions to prevent premature birth may be implemented. Additionally, crosslinking therapies have been successful in treating other tissues such as keratoconus [29] and proposed in tendon [30]. Once we establish the role of crosslinks in women with cervical insufficiency, an application of these therapies has a potential to lead to a reduction in the number of preterm births due to cervical insufficiency. There is a key relationship between cervical collagen changes and mechanical properties in the early softening stage. If these changes can be detected somehow in human pregnancy, this finding can lead to the development of a biomarker for early cervical softening.

### Limitations

The study outlined here is a necessary first step in determining the evolutions of immature and mature crosslinks in normal mouse cervical remodeling and in understanding the relationship between crosslinks and mechanical properties. However, there are limitations to the study. First, we calculated on average stiffer properties for d6 compared to NP samples, which we did not expect. This result could be due to differences in the estrus cycle of the NP mice, which were not taken into account for this study. Further studies utilizing larger sample sizes with cycled NP samples are recommended to confirm statistical significance. Second, only gestation time points on day 6, 12, 15, and 18 were investigated in this study. The short gestation timeline for the mouse provokes the relatively fast process of cervical remodeling in mice. Additional gestational time points, especially between NP and d15 will help understand the rate of collagen turnover during this period. Third, all crosslink measurements presented here were mechanically tested to break before processing for UPLC-ESI-MS/MS. Since tissue samples were previously frozen at 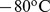 and not metabolically active, we suspect that there are no mechanisms that occur during mechanical testing that can alter the tissue crosslink density. However, additional tests are needed to validate this hypothesis and to confirm that mechanically testing and breaking the tissue does not affect crosslinks measurements. Fourth, the large spread in the crosslink data presented here are most likely due to inconsistency in removing the vaginal wall from mechanically tested samples. The removal of the vaginal wall from the samples was made difficult due to the altered cervix/vaginal structure after breaking the tissue with testing. Finally, the mechanical test conducted in this study is a simple structural tension test to measure the overall structural properties but not its material properties (i.e., stress vs cervical opening instead of strain). Due to the small specimen dimensions and complicated geometry of the cervix, the material properties are difficult to measure. The non-rigid sutures used here to test the cervixes cause some lateral deformation, especially at higher cervical openings. We are currently improving our mechanical test techniques to minimize lateral deformation and developing non-contact techniques based on camera images to measure tissue deformation. Additionally, statistically significant differences in the mechanical properties for samples between d12 to PP cannot be detected with the structural mechanical parameters presented here. Therefore, we are currently improving advanced material modeling techniques to quantify tissue softening in pregnancy.

## Conclusions

In pregnancy, the cervix transforms from a strong closed structure into a soft dilated structure for delivery. In order to understand potential complications resulting from an acceleration or a disruption in this transformation, we investigated the evolution of immature and mature crosslinks in a normal mouse pregnancy and its relationship to tissue mechanical properties. The results presented here show that the maturity of the collagen crosslinks in mouse cervical tissue shift from mature to immature with progressing gestation, indicating a fast turnover of collagens that occur during pregnancy. Additionally, the crosslinks and maturity ratio correlate significantly with tissue mechanical properties. These results demonstrate that in normal cervical remodeling, crosslinks have a role in the early softening process with a reduction in mature crosslinks leading to more compliant and weaker tissue. However, crosslinks do not seem to have a role in further increasing tissue compliance after d15.
